# Ultrasonic flow ratio versus Murray law-based quantitative flow ratio for predicting target vessel failure after IVUS-guided PCI

**DOI:** 10.3389/fcvm.2026.1877973

**Published:** 2026-07-28

**Authors:** Jingao Jiang, Ziyi Yan, Wenhao Huang, Shumin Lv, Xiaochang Leng, Tingting Chen, Wei Mao, Changqing Du

**Affiliations:** 1 The Second School of Clinical Medicine, Zhejiang Chinese Medical University, Hangzhou, China; 2 Department of Cardiology, Affiliated Hospital of Hangzhou Normal University, Hangzhou, China; 3 Zhejiang Chinese Medical University, Hangzhou, China; 4 Hangzhou Daxin Medical Technology Co., Ltd., Hangzhou, China; 5 Zhejiang Key Laboratory of Integrative Chinese and Western Medicine for Diagnosis and Treatment of Circulatory Diseases, Zhejiang Hospital (Affiliated Zhejiang Hospital, Zhejiang University School of Medicine), Hangzhou, Zhejiang, China; 6 Department of Cardiology, Zhejiang Hospital, Hangzhou, China

**Keywords:** coronary artery disease, intravascular ultrasound, Murray law-based quantitative flow ratio, percutaneous coronary intervention, target vessel failure, ultrasonic flow ratio

## Abstract

Residual ischemia after percutaneous coronary intervention (PCI) is closely associated with adverse clinical outcomes. Murray law-based quantitative flow ratio (μQFR), derived from coronary angiography, has demonstrated prognostic value after PCI. Ultrasonic flow ratio (UFR), derived from intravascular ultrasound (IVUS), integrates intravascular imaging with computational physiological assessment; however, its prognostic performance relative to μQFR in patients undergoing IVUS-guided PCI remains insufficiently defined. This retrospective single-center study included 127 patients who underwent IVUS-guided PCI at the Sandun Campus of Zhejiang Hospital between January 2021 and December 2022 and completed follow-up coronary angiography during the follow-up period. UFR and μQFR were measured at the distal exit of the most distal stent. The primary endpoint was target vessel failure (TVF). Receiver operating characteristic curve analysis and Cox proportional hazards regression were performed. Among the 127 included patients, 19 developed TVF and 108 remained free of TVF. Compared with the non-TVF group, the TVF group had significantly lower minimum stent expansion rate, mean stent expansion rate, and post-PCI UFR. UFR showed the highest discriminatory ability for TVF and significantly outperformed μQFR. In multivariable analysis, only post-PCI UFR remained independently associated with TVF. These findings suggest that IVUS-derived anatomical-functional assessment may provide additional value for post-PCI risk stratification.

## 1 Introduction

Coronary artery disease (CAD) remains a leading cause of morbidity and mortality worldwide. Although percutaneous coronary intervention (PCI) improves outcomes in patients undergoing revascularization, adverse events related to residual myocardial ischemia may still occur after the procedure (1). Assessment based on coronary angiography alone may fail to identify residual physiological impairment or suboptimal stent results in some patients. Therefore, accurate post-PCI risk stratification remains clinically important.

Fractional flow reserve (FFR) is an established standard for assessing the functional significance of coronary stenosis and guiding revascularization strategies. Although recommended in current guidelines for the management of coronary artery disease (2, 3), the routine clinical use of FFR remains limited because it is invasive, requires a pressure wire, and depends on vasodilator-induced hyperemia (4, 5). Therefore, non-invasive or wire-free physiological indices may provide practical alternatives for routine post-PCI functional assessment. Recent advances in computational fluid dynamics and artificial intelligence have facilitated the development of wire-free physiological indices derived from coronary imaging. Murray law-based quantitative flow ratio (μQFR), derived from coronary angiography without the need for pressure wires or vasodilators, has emerged as a practical alternative to FFR (6, 7). Its prognostic value after PCI has also been demonstrated in previous studies, in which lower post-PCI QFR or μQFR values were associated with a higher risk of vessel-level adverse outcomes (8–12). For example, the HAWKEYE study identified post-PCI QFR ≤ 0.89 as the optimal cutoff for vessel-oriented composite endpoint, and a *post hoc* analysis of the FLAVOUR trial showed that post-PCI μQFR < 0.90 was associated with a higher risk of 2-year TVF (8, 12). However, μQFR is angiography-derived and may be affected by image quality, vessel overlap, and lesion complexity, potentially limiting its sensitivity for detecting residual functional impairment. Ultrasonic flow ratio (UFR) is an IVUS-derived physiological index that integrates anatomical imaging with computational fluid dynamics to enable combined anatomical and functional assessment of coronary lesions (13, 14). Because it does not require an additional pressure wire or vasodilator agents, UFR may provide a practical alternative for post-PCI physiological evaluation. Previous studies have supported the diagnostic feasibility of IVUS-derived physiological assessment, and emerging evidence suggests that lower post-PCI IVUS-based physiological indices may be associated with subsequent vessel-oriented adverse events (15, 16). For example, a previous study using an IVUS-derived FFR algorithm showed that a lower post-PCI value, with a cutoff of ≤ 0.94, was associated with a higher risk of 2-year vessel-oriented clinical events (16). In addition, the UFR algorithm can automatically generate stent expansion-related parameters during analysis. Large randomized trials, including IVUS-XPL (17) and ULTIMATE (18), have demonstrated that IVUS-guided PCI improves clinical outcomes by optimizing stent implantation. Thus, UFR and μQFR are derived from different imaging modalities and may provide different perspectives on post-PCI vessel assessment. However, in patients undergoing IVUS-guided PCI, the relative prognostic value of post-PCI UFR, μQFR, and stent expansion-related parameters remains insufficiently defined. Therefore, this study aimed to compare the prognostic value of post-PCI UFR, μQFR, and stent expansion-related parameters generated during UFR analysis for predicting target vessel failure (TVF) after IVUS-guided PCI.

## 2 Methods

### 2.1 Study design and population

**Figure 1:**
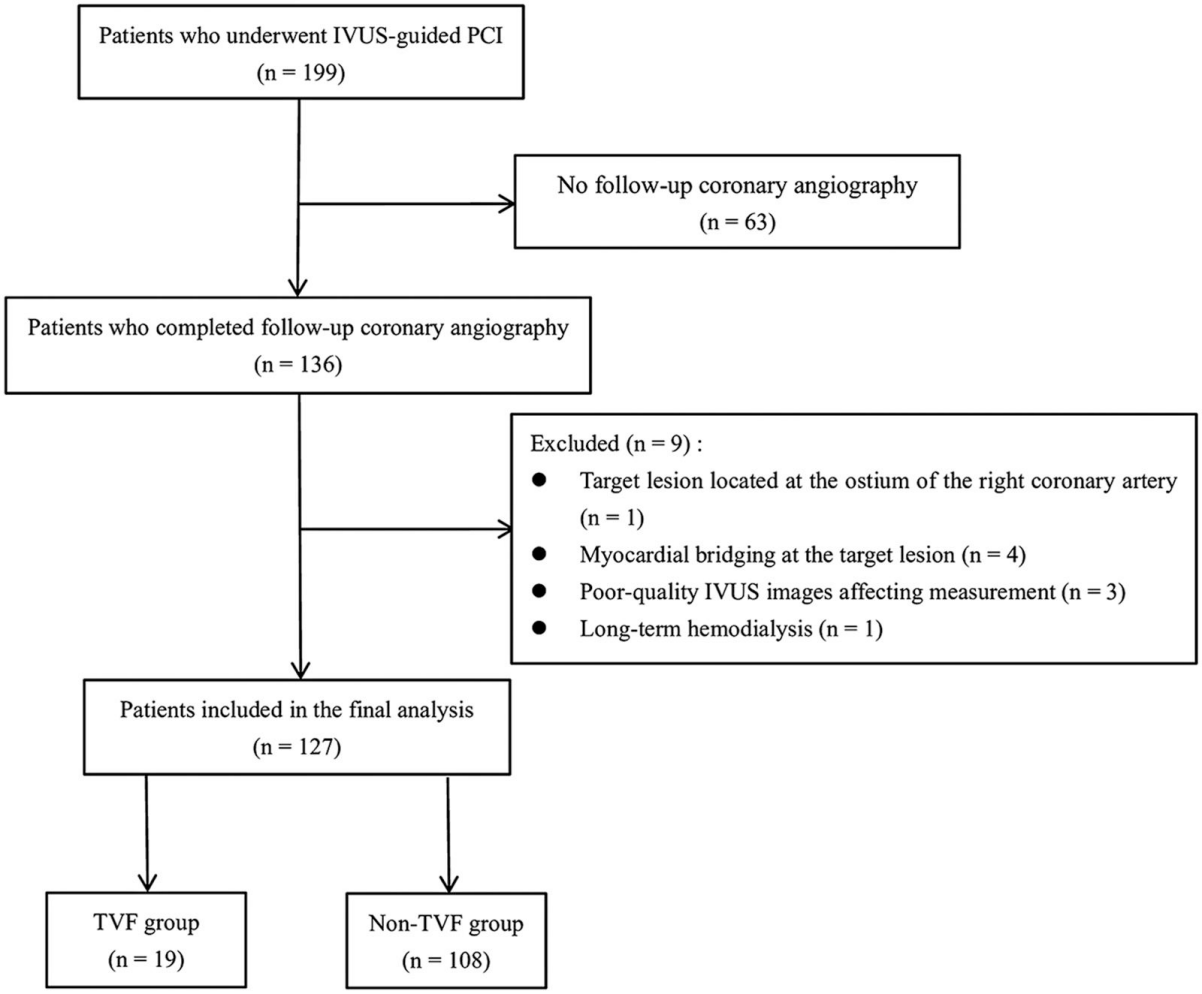
Study flowchart. IVUS, intravascular ultrasound; PCI, percutaneous coronary intervention.

This retrospective, single-center study was conducted at the Sandun Campus of Zhejiang Hospital and screened patients who underwent IVUS-guided PCI between January 2021 and December 2022. Eligibility was determined according to the following inclusion and exclusion criteria. The inclusion criteria were as follows: (1) age ≥ 18 years; (2) coronary artery disease with at least one target lesion treated with IVUS-guided stent implantation; (3) follow-up coronary angiography available after PCI. The exclusion criteria were as follows: (1) prior PCI or coronary artery bypass grafting (CABG) involving the target vessel; (2) target lesions located at the ostium of the left main or right coronary artery; (3) myocardial bridging at the target lesion; (4) poor-quality angiographic images precluding μQFR analysis; (5) poor-quality IVUS images affecting UFR measurement; (6) incomplete IVUS pullback not covering the entire lesion; (7) severe heart failure, significant valvular disease, or long-term hemodialysis. The study flowchart is shown in Figure 1. The study was approved by the Institutional Review Board of Zhejiang Hospital (Approval No. ZJHIRB-2026-011K).The requirement for informed consent was waived because the study used anonymized clinical data, did not involve personally identifiable information, and ensured adequate protection of patient privacy. The study was conducted in accordance with the principles of the Declaration of Helsinki and its later amendments.

### 2.2 Measurement of IVUS-related parameters and UFR

IVUS-related parameters, including plaque burden, number of stents, total stent length, minimum stent area (MSA), minimum stent expansion rate, mean stent expansion rate, and UFR, were analyzed using dedicated software (IvusPlus, Shanghai Bodong Medical Technology Co., Ltd., Shanghai, China). All IVUS examinations were performed using a Boston Scientific/SCIMED intravascular ultrasound system (Minneapolis, MN, USA). UFR calculation consisted of two major steps: geometric reconstruction and pressure-drop computation along the IVUS pullback. First, the lumen contours and external elastic lamina (EEL) were automatically delineated using a deep-learning model and reconstructed in three dimensions. Manual correction was allowed when the automatically delineated contours did not accurately follow the lumen or EEL borders. Second, the ostia of side branches perpendicular to the side-branch centerlines were reconstructed, and side-branch areas were quantified. Finally, the pressure drop at each cross-section along the pullback was calculated using a validated computational FFR method based on fluid dynamic equations, thereby generating the UFR pullback (14). The IVUS pullback was required to cover the entire lesion. For post-PCI physiological assessment, the distal measurement point was defined as the distal exit of the most distal stent. The proximal boundary corresponded to the coronary ostium reconstructed from the IVUS pullback. Thus, the UFR value reflected the physiological characteristics of the interrogated vessel segment from the coronary ostium to the distal stent edge.

### 2.3 Stent expansion parameters

**Figure 2:**
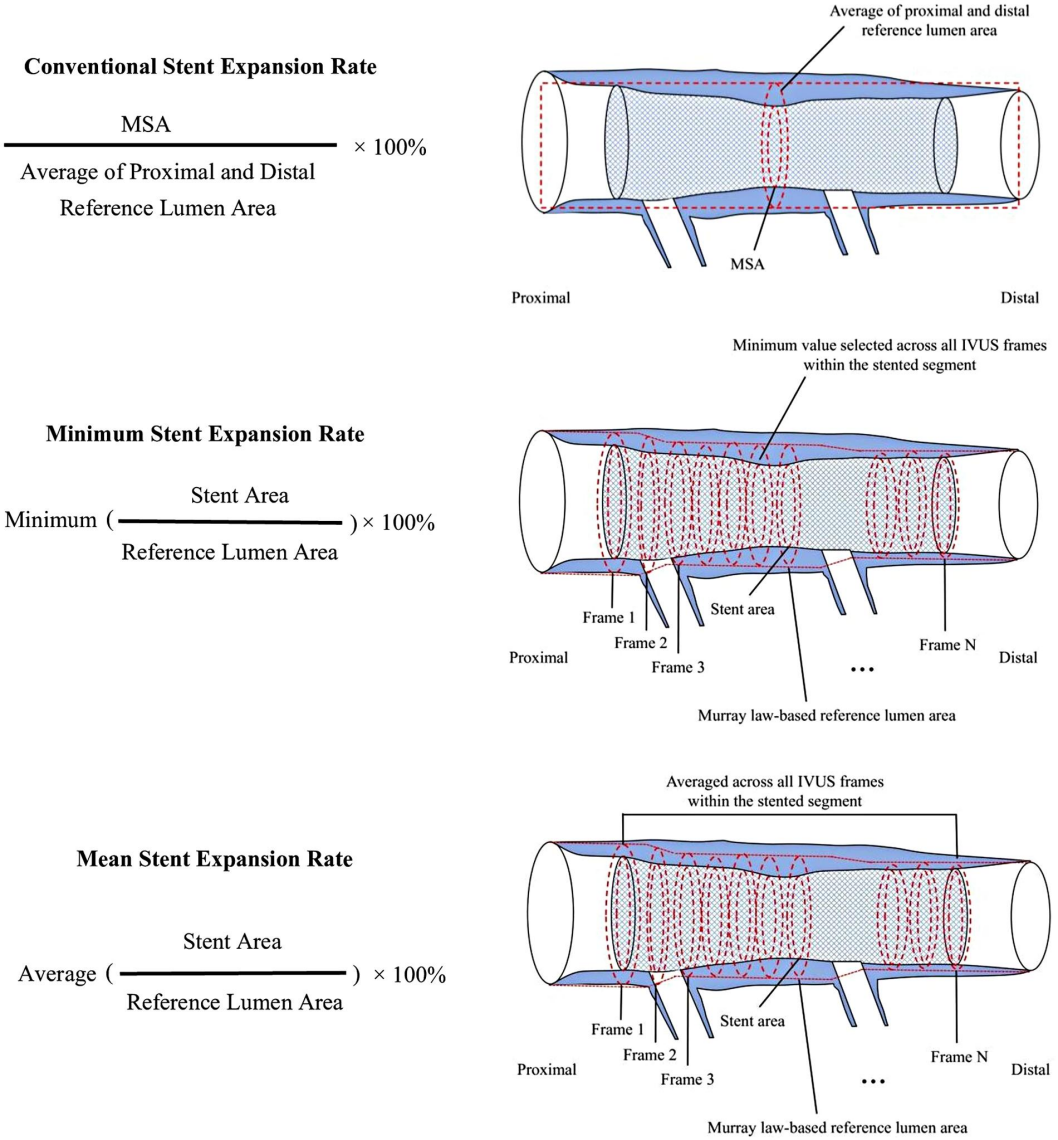
Schematic illustration of stent expansion parameters. Conventional stent expansion is shown as a reference definition and is commonly calculated as MSA divided by the average of proximal and distal reference lumen areas. The minimum stent expansion rate was defined as the minimum value of the frame-level ratio of stent area to the corresponding reference lumen area across all IVUS frames within the stented segment. The reference lumen area at each frame was generated by the software based on Murray law. The mean stent expansion rate was defined as the arithmetic mean of these frame-level ratios. Both parameters were expressed as percentages. The dashed contours indicate the reference lumen areas generated by the software rather than observed lumen contours. MSA, minimum stent area; IVUS, intravascular ultrasound.

During UFR analysis, the algorithm also generated stent expansion-related parameters, including the minimum stent expansion rate and the mean stent expansion rate. The minimum stent expansion rate was defined as the minimum value of the frame-level ratio of stent area to the corresponding reference lumen area across all IVUS frames within the stented segment. The reference lumen area at each frame was generated by the software based on Murray law. The mean stent expansion rate was defined as the arithmetic mean of these frame-level ratios. Both parameters were expressed as percentages. These stent expansion parameters generated during UFR analysis differ from conventional reference-segment-based stent expansion, which is commonly calculated as MSA divided by the average of the proximal and distal reference lumen areas. Conventional stent expansion is shown in Figure 2 as a reference definition to clarify differences among the expansion parameters.

### 2.4 Measurement of μQFR

**Figure 3:**
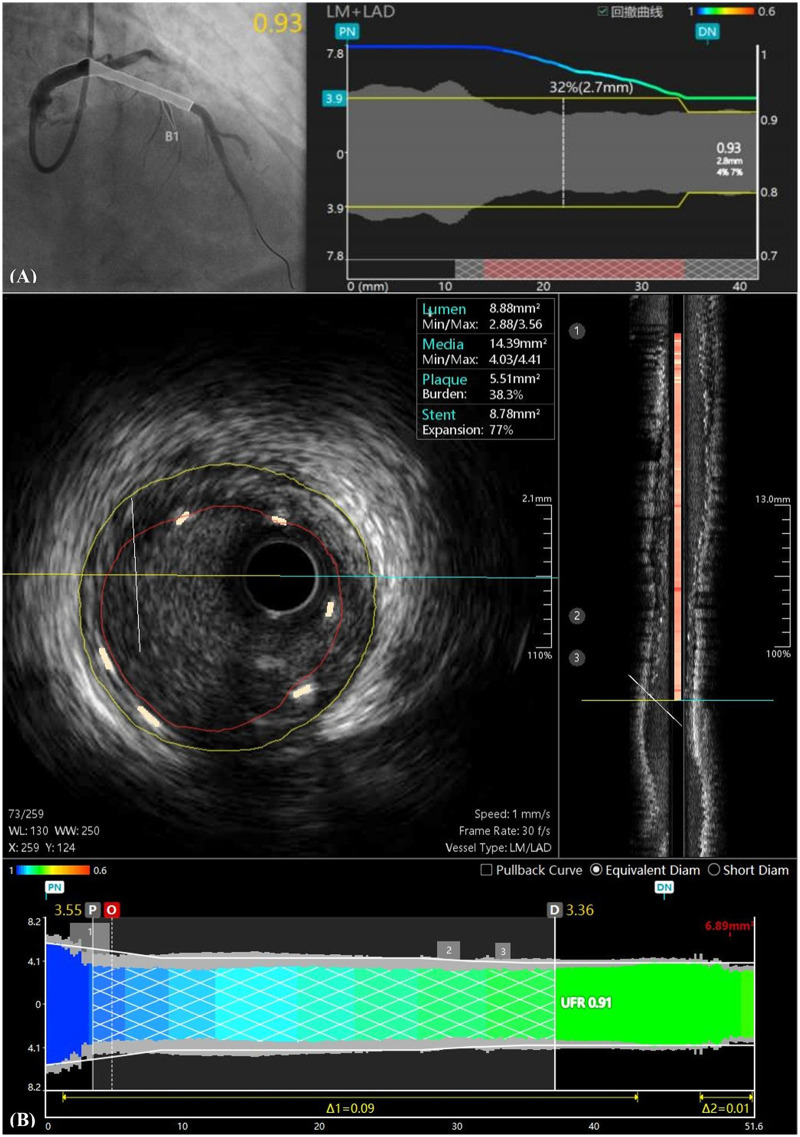
Representative example of post-PCI μQFR and UFR measurements. **(A)** μQFR measured at the distal exit of the most distal stent = 0.93. **(B)** UFR measured at the distal exit of the most distal stent = 0.91. μQFR, Murray law-based quantitative flow ratio; UFR, ultrasonic flow ratio.

μQFR analysis was performed by a blinded analyst using a prototype software package (AngioPlus Core software, Pulse Medical Imaging Technology, Shanghai, China). μQFR was calculated using an artificial intelligence-based algorithm that enabled single-view semi-automated vessel contour delineation and FFR simulation. First, a key frame was selected from the optimal angiographic view in which the luminal contour at the stenotic segment was clearly visible. The vessel contour was then automatically delineated, and the reference lumen diameter was reconstructed according to the principles of coronary flow distribution. The proximal and distal reference lumen diameters could be manually adjusted when necessary. In cases of severe stenosis or automated misidentification, manual correction was performed to ensure accuracy. Finally, μQFR was computed for the target vessel (19). To ensure consistency with UFR measurement, the μQFR measurement point was defined as the distal exit of the most distal stent. Figure 3 shows a representative example of UFR and μQFR computation.

### 2.5 Endpoint and follow-up

The primary endpoint was TVF, defined according to the ARC-2 criteria as a composite of cardiac death, target vessel-related myocardial infarction, and target vessel revascularization (20). Cardiac death was defined as death due to any cardiac event, including myocardial infarction, arrhythmia, heart failure, or cardiac arrest. A target vessel-related myocardial infarction was defined as recurrent myocardial infarction after PCI involving the target vessel, diagnosed by elevated cardiac biomarkers together with ischemic evidence, such as electrocardiographic changes or imaging evidence of new loss of viable myocardium or new regional wall motion abnormality. Target vessel revascularization (TVR) was defined as an unplanned repeat PCI or CABG procedure related to the target vessel after PCI. Follow-up was censored at the first occurrence of TVF or at the time of the first follow-up coronary angiography in patients without TVF. Follow-up coronary angiography was performed according to routine clinical practice and physician judgment and was not mandated by the study protocol. All endpoint events were adjudicated by two independent investigators blinded to UFR and μQFR measurements, and disagreements were resolved by a third senior investigator.

### 2.6 Statistical analysis

Continuous variables were expressed as mean ± standard deviation or median with interquartile range, as appropriate, and categorical variables were presented as counts and percentages. Patients were classified into TVF and non-TVF groups according to follow-up outcomes. Between-group comparisons were performed using Pearson's chi-squared test, Fisher's exact test, unpaired Student's t-test, or the Mann–Whitney U test, as appropriate. Receiver operating characteristic (ROC) curves were constructed to evaluate the discriminatory performance of UFR, μQFR, MSA, minimum stent expansion rate, and mean stent expansion rate for TVF after PCI. The area under the ROC curve (AUC) was calculated, and DeLong's test was used to compare differences in AUC between parameters. Univariable and multivariable Cox proportional hazards regression models were used to identify predictors of TVF. Because only 19 TVF events occurred in the study cohort, the number of variables included in the multivariable Cox model was intentionally restricted to reduce the risk of overfitting, with consideration of the limited events-per-variable ratio. The multivariable model therefore included only the post-PCI parameters that were significant in univariable analysis and were most relevant to the primary study objective. Accordingly, the multivariable results should be interpreted as exploratory. Because post-PCI UFR, μQFR, and mean stent expansion rate all reflect the post-PCI status of the treated vessel and may be biologically interrelated, the multivariable model was intended as an exploratory analysis to compare their relative associations with TVF and to assess which post-PCI parameter might be most informative for risk stratification, rather than to establish complete statistical independence or causal incremental value. As a sensitivity analysis for rare events, Firth penalized Cox regression was additionally performed using the same covariates included in the primary multivariable model. Statistical analyses were performed using SPSS (version 25.0) and R (version 4.4.1). A two-sided *p* value <0.05 was considered statistically significant.

## 3 Results

### 3.1 Baseline characteristics

Between January 2021 and December 2022, a total of 199 patients underwent IVUS-guided PCI at the Sandun Campus of Zhejiang Hospital. Among them, 63 patients did not undergo follow-up coronary angiography, and 136 patients completed follow-up coronary angiography during the follow-up period. According to the predefined exclusion criteria, 9 patients were excluded, including 1 with a target lesion located at the ostium of the right coronary artery, 4 with myocardial bridging at the target lesion, 3 with poor-quality IVUS images affecting measurement, and 1 receiving long-term hemodialysis. Ultimately, 127 patients were included in the final analysis, of whom 19 experienced TVF during follow-up (Figure 1). The median follow-up duration was 12.000 months [interquartile range, 12.000–15.000 months] in the overall cohort, with no significant difference between the TVF and non-TVF groups (13.000 [12.000–18.000] vs. 12.000 [12.000–15.000] months; *p* = 0.813; Table 1). Among the 19 TVF events, all patients underwent target vessel revascularization, 3 patients also had target vessel-related myocardial infarction, and no cardiac deaths occurred. Among these patients, the TVR leading to TVF was associated with documented recurrent chest tightness or angina-like symptoms in 10 patients, whereas TVR was identified during follow-up coronary angiography in 9 patients without clearly documented preceding ischemic symptoms. Baseline characteristics were generally similar between the two groups, with no significant differences in demographic characteristics, cardiovascular risk factors, clinical presentation, medical history, laboratory findings, or discharge medications (all *p* > 0.05; Table 1).

**Table 1:** Baseline demographic and clinical characteristics.

Variable	Total (*N* = 127)	TVF Group (*n* = 19)	Non-TVF Group (*n* = 108)	*p* value
Follow-up duration, months	12.000 [12.000–15.000]	13.000 [12.000–18.000]	12.000 [12.000–15.000]	0.813
Demographics				
Age, years	62.000 [55.000–68.000]	65.000 [59.000–68.000]	61.500 [54.000–68.750]	0.313
Male, *n* (%)	101 (79.5)	15 (79.0)	86 (79.6)	0.946
BMI, kg/m^2^	24.390 [22.260–27.310]	24.640 [22.720–26.640]	24.390 [22.250–27.360]	0.556
Cardiovascular risk factors				
Hypertension, *n* (%)	82 (64.6)	16 (84.2)	66 (61.1)	0.052
Diabetes, *n* (%)	38 (29.9)	6 (31.6)	32 (29.6)	0.864
Dyslipidemia, *n* (%)	16 (12.6)	4 (21.1)	11 (10.2)	0.065
Smoking history, *n* (%)	63 (49.6)	7 (36.9)	56 (51.9)	0.229
Alcohol consumption history, *n* (%)	42 (33.1)	5 (26.3)	37 (34.3)	0.499
Clinical presentation				
CCS, *n* (%)	40 (31.5)	5 (26.3)	35 (32.4)	0.598
ACS, *n* (%)	87 (68.5)	14 (73.7)	73 (67.6)	0.598
Medical history				
Prior CABG (non-target vessel), *n* (%)	1 (0.8)	0 (0.0)	1 (0.9)	0.675
Prior PCI (non-target vessel), *n* (%)	21 (16.5)	5 (26.3)	16 (14.8)	0.215
Laboratory parameters				
TG, mmol/L	1.410 [1.040–1.990]	1.400 [0.960–2.410]	1.420 [1.040–1.930]	0.415
HDL-C, mmol/L	1.003 ± 0.020	0.987 ± 0.187	1.006 ± 0.231	0.858
LDL-C, mmol/L	2.420 [1.670–3.170]	2.560 [1.760–3.040]	2.415 [1.648–3.253]	0.233
LVEF, %	66.100 [59.800–70.300]	67.300 [63.700–69.900]	65.550 [59.025–70.500]	0.598
HbA1c, %	6.100 [5.700–6.900]	6.300 [5.900–7.000]	6.100 [5.700–6.900]	0.183
Creatinine, μmol/L	76.000 [66.000–88.000]	75.000 [65.000–85.000]	76.500 [67.250–88.750]	0.778
Discharge medications				
Aspirin, *n* (%)	126 (99.2)	19 (100)	107 (99.1)	0.674
Ticagrelor, *n* (%)	40 (31.5)	5 (26.3)	35 (32.4)	0.598
Clopidogrel, *n* (%)	86 (67.7)	14 (73.7)	72 (66.7)	0.546
Statins, *n* (%)	124 (97.6)	18 (94.7)	106 (98.2)	0.367

Continuous variables are presented as mean ± standard deviation or median [interquartile range], as appropriate, and categorical variables are presented as *n* (%). Follow-up duration was defined as the time from index PCI to the occurrence of TVF in the TVF group, or to the date of the first follow-up coronary angiography in the non-TVF group.

TVF, target vessel failure; BMI, body mass index; CCS, chronic coronary syndrome; ACS, acute coronary syndrome; CABG, coronary artery bypass grafting; PCI, percutaneous coronary intervention; TG, triglyceride; HDL-C, high-density lipoprotein cholesterol; LDL-C, lowdensity lipoprotein cholesterol; LVEF, left ventricular ejection fraction; HbA1c, glycated hemoglobin.

### 3.2 Post-PCI angiographic, stent expansion-related, and physiological characteristics

No significant differences were observed between the TVF and non-TVF groups in target vessel distribution or the prevalence of two-vessel and three-vessel disease (all *p* > 0.05). Among post-PCI stent expansion-related parameters, both the minimum stent expansion rate and the mean stent expansion rate were significantly lower in the TVF group than in the non-TVF group (49.530 ± 15.710% vs. 57.540 ± 12.894%, *P* = 0.017; 75.684 ± 16.439% vs. 88.380 ± 15.517%, *p* = 0.001, respectively). In terms of physiological assessment, post-PCI UFR was significantly lower in the TVF group (0.885 ± 0.060 vs. 0.935 ± 0.037, *p* < 0.001), whereas post-PCI μQFR was numerically lower in the TVF group but did not reach statistical significance (0.934 ± 0.045 vs. 0.955 ± 0.034, *p* = 0.068). Detailed parameters are presented in Table 2.

**Table 2:** Comparison of post-PCI angiographic, stent expansion-related, and physiological parameters between the TVF and non-TVF groups.

Variable	Total (*n* = 127)	TVF group (*n* = 19)	Non-TVF group (*n* = 108)	*p* value
Angiographic parameters				
Target vessel				
LAD	73 (57.5)	10 (52.6)	63 (58.3)	0.643
LCX	22 (17.3)	1 (5.3)	21 (19.4)	0.239
RCA	32 (25.2)	8 (42.1)	24 (22.2)	0.066
Two-vessel disease	43 (33.9)	6 (31.6)	37 (34.3)	0.820
Three-vessel disease	54 (42.5)	9 (47.4)	45 (41.7)	0.643
Stent parameters				
Number of stents	1.000 [1.000–2.000]	2.000 [1.000–2.000]	1.000 [1.000–2.000]	0.072
Total stent length, mm	33.000 [24.000–54.000]	48.000 [29.000–60.000]	31.000 [24.000–52.000]	0.076
MSA, mm2	5.770 [4.360–6.850]	4.920 [3.390–7.050]	5.860 [4.435–6.848]	0.312
Minimum stent expansion rate, %	56.340 ± 13.589	49.530 ± 15.710	57.540 ± 12.894	0.017
Mean stent expansion rate, %	86.480 ± 16.240	75.684 ± 16.439	88.380 ± 15.517	0.001
Physiological parameters				
Post-PCI μQFR	0.952 ± 0.037	0.934 ± 0.045	0.955 ± 0.034	0.068
Post-PCI UFR	0.927 ± 0.045	0.885 ± 0.060	0.935 ± 0.037	<0.001

Continuous variables are presented as mean ± standard deviation or median [interquartile range], as appropriate, and categorical variables are presented as *n* (%).

LAD, left anterior descending artery; LCX, left circumflex artery; RCA, right coronary artery; MSA, minimum stent area; UFR, ultrasonic flow ratio; μQFR, Murray law-based quantitative flow ratio.

### 3.3 Comparative discriminatory performance of post-PCI UFR, μQFR, and stent expansion-related parameters for TVF

**Figure 4:**
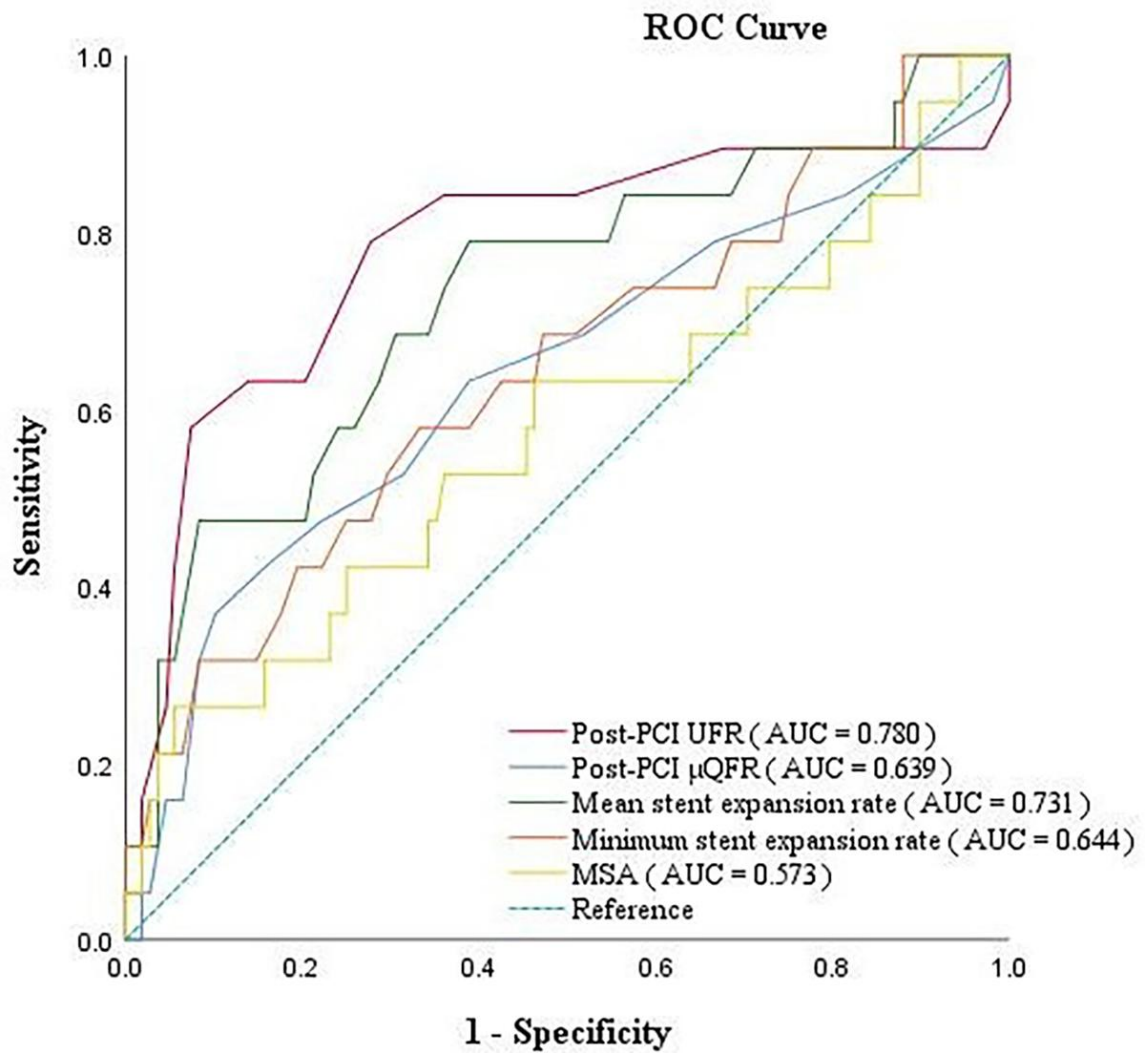
ROC curves for predicting TVF using post-PCI UFR, μQFR, and stent expansion-related parameters. TVF, target vessel failure; PCI, percutaneous coronary intervention; UFR, ultrasonic flow ratio; μQFR, Murray law-based quantitative flow ratio; MSA, minimum stent area.

ROC curve analysis (Figure 4, Table 3) showed that post-PCI UFR had the highest AUC among the evaluated parameters for predicting TVF (AUC = 0.780; 95% CI: 0.640−0.920). Using the Youden index, the exploratory optimal cutoff value for post-PCI UFR was 0.915, corresponding to a sensitivity of 78.9% and a specificity of 72.2% (Supplementary Table S1). In comparison, post-PCI μQFR showed lower discriminatory ability (AUC = 0.639; 95% CI: 0.487–0.791). Among the stent expansion-related parameters, the mean stent expansion rate showed acceptable discriminatory performance (AUC = 0.731; 95% CI: 0.597–0.865; *p* = 0.001), whereas the minimum stent expansion rate showed only modest discriminatory performance (AUC = 0.644; 95% CI: 0.501–0.788; *p* = 0.045). MSA did not show significant discriminatory ability for TVF (AUC = 0.573; 95% CI: 0.417–0.729; *p* = 0.312). DeLong's test showed that UFR significantly outperformed μQFR (*p* = 0.031) and MSA (*p* = 0.001), whereas the difference between UFR and the mean stent expansion rate was not statistically significant (*p* = 0.487; Table 4).

**Table 3:** Discriminatory performance of individual parameters for predicting TVF.

Parameter	AUC	95% CI	*p* value
Post-PCI UFR	0.780	0.640–0.920	<0.001
Post-PCI μQFR	0.639	0.487–0.791	0.054
Mean stent expansion rate	0.731	0.597–0.865	0.001
Minimum stent expansion rate	0.644	0.501–0.788	0.045
MSA	0.573	0.417–0.729	0.312

AUC, area under the receiver operating characteristic curve; CI, confidence interval; UFR, ultrasonic flow ratio; μQFR, Murray law-based quantitative flow ratio; MSA, minimum stent area.

**Table 4:** Pairwise comparison of AUCs between UFR and selected parameters using DeLong's test.

Comparison	AUC difference	95% CI for the difference	*p* value
UFR vs. μQFR	0.142	0.013–0.270	0.031
UFR vs. Mean stent expansion rate	0.049	−0.089–0.186	0.487
UFR vs. MSA	0.210	0.081–0.338	0.001

AUC, area under the receiver operating characteristic curve; CI, confidence interval; UFR, ultrasonic flow ratio; μQFR, Murray law-based quantitative flow ratio; MSA, minimum stent area.

### 3.4 Cox regression analysis of predictors of TVF

Univariable Cox regression analysis (Table 5) showed that higher post-PCI UFR, analyzed per 0.1 increase, was associated with a lower risk of TVF (HR 0.168, 95% CI: 0.063–0.445; *p* < 0.001). Higher post-PCI μQFR, also analyzed per 0.1 increase, was similarly associated with a lower risk of TVF (HR 0.327, 95% CI: 0.121–0.884; *p* = 0.028), as was a higher mean stent expansion rate (HR 0.962, 95% CI: 0.929–0.996; *p* = 0.027). In the multivariable Cox regression model including post-PCI UFR, post-PCI μQFR, and mean stent expansion rate, only post-PCI UFR remained independently associated with TVF (HR 0.147, 95% CI: 0.025–0.856; *p* = 0.033). To evaluate the robustness of the findings given the limited number of TVF events, a Firth penalized Cox regression was performed as a sensitivity analysis (Supplementary Table S2). The results were consistent with the primary multivariable Cox model, with post-PCI UFR remaining significantly associated with TVF (HR 0.135, 95% CI: 0.023–0.828; *p* = 0.030), whereas post-PCI μQFR and mean stent expansion rate were not significant.

**Table 5:** Univariable and multivariable Cox regression analysis for selected predictors of TVF.

	Univariable analysis			Multivariable analysis		
Variable	HR	95%CI	*p* value	HR	95%CI	*p* value
Clinical variables						
Age, years	0.981	0.935–1.030	0.440			
Hypertension	2.458	0.702–8.611	0.160			
Diabetes	0.990	0.373–2.623	0.984			
Post-PCI parameters						
Number of stents	1.163	0.680–1.989	0.582			
Total stent length, mm	1.004	0.988–1.020	0.649			
MSA, mm^2^	0.845	0.649–1.101	0.214			
Minimum stent expansion rate, %	0.969	0.935–1.004	0.079			
Mean stent expansion rate, %	0.962	0.929–0.996	0.027	0.992	0.951–1.036	0.724
Post-PCI μQFR (per 0.1 increase)	0.327	0.121–0.884	0.028	1.405	0.297–6.636	0.668
Post-PCI UFR (per 0.1 increase)	0.168	0.063–0.445	<0.001	0.147	0.025–0.856	0.033

Multivariable analysis included mean stent expansion rate, post-PCI μQFR, and post-PCI UFR.

MSA, minimum stent area; PCI, percutaneous coronary intervention; TVF, target vessel failure; μQFR, Murray law-based quantitative flow ratio; UFR, ultrasonic flow ratio.

## 4 Discussion

This study compared the prognostic value of post-PCI UFR, μQFR, and stent expansion-related parameters generated during UFR analysis for TVF after IVUS-guided PCI. The main findings were as follows. First, post-PCI UFR showed the highest AUC among the evaluated parameters and significantly outperformed μQFR in discriminating TVF (0.780 vs. 0.639; DeLong *p* = 0.031). Although its AUC was numerically higher than that of the mean stent expansion rate, this difference was not statistically significant. Second, in univariable Cox regression analysis, both higher post-PCI UFR and higher post-PCI μQFR were associated with a lower risk of TVF. However, in the multivariable model including post-PCI UFR, post-PCI μQFR, and mean stent expansion rate, only UFR remained independently associated with TVF. Third, the stent expansion-related parameters automatically generated during UFR analysis also showed prognostic relevance, particularly the mean stent expansion rate, which demonstrated acceptable discriminatory performance and was associated with lower TVF risk in univariable analysis. Overall, these findings suggest that post-PCI UFR and the mean stent expansion rate may both be clinically informative for post-PCI risk stratification.

μQFR and UFR are both pressure wire-free physiological indices developed to estimate FFR-equivalent functional information. Post-PCI FFR has been extensively investigated for predicting adverse cardiovascular outcomes, including TVF (21–27). Although FFR remains an invasive reference standard for assessing the functional significance of coronary stenosis, its routine clinical use is limited by the need for a pressure wire and pharmacologically induced hyperemia, which increase procedural complexity and may lead to vasodilator-related adverse effects (28). By avoiding both pressure wires and adenosine, μQFR and UFR may mitigate these limitations and represent practical alternatives for post-PCI physiological assessment. Beyond epicardial coronary physiology, recent studies have also extended angiography-derived computational indices to the evaluation of coronary microvascular function. Zhang et al. (29) reported that a single-view angiography-derived microcirculatory resistance index was associated with coronary microvascular dysfunction and adverse clinical outcomes after primary PCI, supporting the broader role of wire-free physiological assessment in post-procedural risk stratification.

μQFR is derived from coronary angiography through lumen reconstruction and flow simulation to estimate an FFR-equivalent pressure ratio (6). Previous studies have shown that a lower post-PCI μQFR is associated with a higher risk of adverse clinical outcomes. Aurigemma et al. (11) reported a significantly higher incidence of TVF in patients with lower post-PCI μQFR, and Ding et al. (12) reported similar findings. In the present study, higher post-PCI μQFR was also associated with a lower risk of TVF in univariable analysis. However, its overall discriminatory performance was modest (AUC = 0.639), and it did not remain independently associated with TVF in the multivariable model. Several factors may explain this finding. As an angiography-derived physiological index, μQFR may be influenced by image quality, vessel overlap, tortuosity, contrast filling, and lesion complexity. In addition, angiography alone cannot fully characterize vessel wall morphology, plaque burden, or stent expansion status after PCI. These limitations may reduce the sensitivity of μQFR for detecting residual functional impairment after PCI (30). Furthermore, angiographic assessment is inherently limited by the two-dimensional representation of a three-dimensional vessel structure. Vessel eccentricity, overlap, and foreshortening may further affect lumen reconstruction and physiological estimation. Consistent with these considerations, the REPEAT-QFR study (31) demonstrated that variability in angiography-derived physiological measurements was influenced by angiographic image quality and procedural analysis quality, highlighting the importance of standardized image acquisition and analysis when interpreting μQFR measurements.

UFR is an IVUS-derived physiological index that enables simultaneous anatomical and functional assessment of coronary lesions without the need for an additional pressure wire or vasodilator agents (14). Previous studies have shown that IVUS-guided PCI improves clinical outcomes compared with angiography-guided PCI, and current guidelines also recommend IVUS for optimizing stent implantation in appropriate clinical and anatomical settings (2, 17, 18). By integrating physiological computation with intravascular imaging findings, UFR may provide a more comprehensive assessment of residual functional impairment after PCI. Yang et al. reported the diagnostic performance of UFR for identifying functionally significant coronary stenosis (32). In addition, Huang et al. used another IVUS-based physiological algorithm, AccuFFRivus, and found that lower post-PCI values were associated with a higher incidence of vessel-oriented adverse events during follow-up (16). Similarly, in the present study, UFR showed favorable discriminatory performance for TVF and significantly outperformed μQFR.

From the perspective of stent optimization, comparison between standard post-PCI expansion assessment and more complex computed physiological indices is clinically relevant. Absolute MSA has been the most commonly studied IVUS-derived stent optimization parameter, and prior studies have supported its association with clinical outcomes after PCI (33). However, the prognostic value of different stent expansion indexes has not been entirely consistent. In the ADAPT-DES IVUS substudy (34), 10 stent expansion indexes were evaluated, including absolute MSA, conventional stent expansion, and model-based expansion indexes. Among these indexes, only MSA divided by the vessel area at the MSA site was independently associated with 2-year clinically driven target lesion revascularization or definite stent thrombosis after adjustment for morphologic and procedural parameters, whereas several other expansion indexes were not independently associated with outcomes. These findings suggest that relative expansion assessment may provide prognostic information that is not fully captured by absolute MSA or conventional reference-segment-based expansion alone. In line with this concept of relative expansion assessment, the present study further evaluated two Murray law-based relative stent expansion parameters generated during UFR analysis: the minimum stent expansion rate and the mean stent expansion rate. The minimum stent expansion rate reflected the lowest frame-level expansion relative to the reference lumen area within the stented segment. By contrast, the mean stent expansion rate characterized the average frame-level expansion relative to the reference lumen area across the entire stented segment. In our study, absolute MSA was numerically lower in the TVF group but was not significantly associated with TVF. Both the minimum and mean stent expansion rates were lower in patients with TVF and showed significant discriminatory ability; however, the mean stent expansion rate demonstrated stronger discriminatory performance than the minimum stent expansion rate. Importantly, the discriminatory performance of the mean stent expansion rate was not significantly different from that of UFR in ROC analysis. The mean stent expansion rate was also associated with lower TVF risk in univariable Cox analysis, suggesting that average relative expansion across the stented segment may provide complementary information to the single lowest-expansion cross-section. However, the mean stent expansion rate did not remain significant after adjustment. In contrast, only UFR remained associated with TVF in the exploratory multivariable model. Given that the discriminatory performance of UFR and the mean stent expansion rate was not significantly different, this finding should be interpreted cautiously. Therefore, the present data do not establish definitive incremental prognostic value of UFR beyond stent expansion assessment. Rather, they suggest that post-PCI UFR and stent expansion-related parameters may provide complementary information, with UFR potentially reflecting the functional consequence of the treated vessel segment in addition to the mechanical result of stent implantation.

Direct comparative studies evaluating the prognostic value of post-PCI UFR and μQFR remain limited. Zhao et al. investigated post-PCI UFR and μQFR in patients undergoing rotational atherectomy followed by stent implantation and found that the two parameters showed comparable performance for predicting 12-month major adverse cardiovascular events (35). Several differences may explain the discrepancy between their findings and ours. First, patients undergoing rotational atherectomy usually have severely calcified lesions and represent a highly selected procedural subgroup, whereas the present study evaluated patients undergoing IVUS-guided PCI in a routine clinical setting. Second, Zhao et al. used major adverse cardiovascular events as the endpoint, whereas our study focused on TVF, a target vessel-specific outcome that may be more directly related to residual vessel-level physiological impairment after PCI. Furthermore, a relevant methodological feature of the present study was that the measurement location for both UFR and μQFR was standardized at the distal exit of the most distal stent, ensuring that the two indices were assessed at the same anatomical site. This approach may have improved the comparability of the two physiological measurements and strengthened the evaluation of their relative prognostic performance.

This study has several limitations. First, it was a single-center retrospective study with a relatively small sample size and a limited number of TVF events. Therefore, the present study should be regarded as exploratory and hypothesis-generating. Although the number of variables included in the multivariable Cox model was intentionally restricted to reduce overfitting, the limited event count may have reduced statistical power and affected model stability. To address this concern, a Firth penalized Cox regression was performed as a sensitivity analysis, and the results remained consistent with those of the primary multivariable model. The exploratory post-PCI UFR cutoff should be interpreted cautiously due to the limited sample size and number of events, and is presented for exploratory purposes rather than as a definitive clinical threshold. Nevertheless, the findings should be validated in larger prospective multicenter cohorts. Second, only patients who underwent follow-up coronary angiography after IVUS-guided PCI were included, which may have introduced selection bias. To evaluate this issue, baseline characteristics were compared between the final study cohort and patients excluded because follow-up coronary angiography was unavailable (Supplementary Table S3), and most baseline demographic and clinical characteristics were comparable between the two groups. This issue is particularly relevant because TVF events in this cohort were predominantly driven by target vessel revascularization, and repeat angiography may have influenced the detection and treatment of restenosis or residual disease. Among the 19 patients with TVF, the TVR leading to TVF was associated with clinical symptoms in 10 patients, whereas the TVR was identified during follow-up coronary angiography in 9 patients. Therefore, although an angiography-driven component cannot be completely excluded, not all TVF events were detected solely through routine angiographic surveillance. Nevertheless, selection bias related to follow-up angiography and angiography-driven event ascertainment should be considered when interpreting the prognostic findings. Third, although follow-up duration did not differ significantly between the TVF and non-TVF groups, follow-up coronary angiography was performed at non-uniform time intervals across patients, which may have introduced heterogeneity in event ascertainment. Finally, because both UFR and μQFR reflect the physiological characteristics of the interrogated vessel rather than the treated lesion alone, residual non-critical disease within the target vessel may have influenced post-PCI physiological measurements. The potential impact of this factor was not separately evaluated in the present study.

## 5 Conclusions

In patients undergoing IVUS-guided PCI, post-PCI UFR showed the strongest overall performance for predicting TVF among the evaluated parameters and remained independently associated with TVF after adjustment. Stent expansion-related parameters generated during UFR analysis, particularly the mean stent expansion rate, also showed prognostic relevance. By integrating intravascular imaging with computational physiological assessment, UFR may provide additional value for post-PCI risk stratification.

## Data Availability

The raw data supporting the conclusions of this article will be made available by the authors, without undue reservation.
