# Supplementary material for: Ultrasonic flow ratio versus Murray law-based quantitative flow ratio for predicting target vessel failure after IVUS-guided PCI
Source: Front Cardiovasc Med. 2026 Jul 28;13:1877973. doi: 10.3389/fcvm.2026.1877973 (PMC13457620; doi:10.3389/fcvm.2026.1877973)
Supplement: Supplementary file 1 [file Table1.docx]

**Supplementary Table S1. Exploratory optimal cutoff value of post-PCI UFR for predicting TVF**

| Variable | AUC (95% CI) | Cutoff | Sensitivity (%) | Specificity (%) | Youden index |
| --- | --- | --- | --- | --- | --- |
| Post-PCI UFR | 0.780 (0.640 - 0.920) | 0.915 | 78.9 | 72.2 | 0.511 |

The cutoff was determined using the Youden index. Sensitivity, specificity, and Youden index values are shown. PCI, percutaneous coronary intervention; UFR, ultrasonic flow ratio; TVF, target vessel failure.
