# Supplementary material for: Ultrasonic flow ratio versus Murray law-based quantitative flow ratio for predicting target vessel failure after IVUS-guided PCI
Source: Front Cardiovasc Med. 2026 Jul 28;13:1877973. doi: 10.3389/fcvm.2026.1877973 (PMC13457620; doi:10.3389/fcvm.2026.1877973)
Supplement: Supplementary file 2 [file Table2.docx]

**Supplementary Table S2 Sensitivity analysis using Firth penalized Cox regression**

| **Variable** | HR | 95% CI | *P* value |
| --- | --- | --- | --- |
| Mean stent expansion rate, % | 0.966 | 0.951-1.037 | 0.838 |
| Post-PCI μQFR (per 0.1 increase) | 1.430 | 0.308-7.105 | 0.652 |
| Post-PCI UFR (per 0.1 increase) | 0.135 | 0.023-0.828 | 0.030 |

Sensitivity analysis of post-PCI predictors of TVF using Firth penalized Cox regression. PCI, percutaneous coronary intervention; μQFR, Murray law-based quantitative flow ratio; UFR, ultrasonic flow ratio; TVF, target vessel failure.
