# Supplementary material for: Ultrasonic flow ratio versus Murray law-based quantitative flow ratio for predicting target vessel failure after IVUS-guided PCI
Source: Front Cardiovasc Med. 2026 Jul 28;13:1877973. doi: 10.3389/fcvm.2026.1877973 (PMC13457620; doi:10.3389/fcvm.2026.1877973)
Supplement: Supplementary file 3 [file Table3.docx]

**Supplementary Table S3. Comparison of baseline characteristics between included patients and patients excluded because of unavailable follow-up coronary angiography.**

| **Variable** | Included (n = 127) | Excluded (n = 63) | *p* value |
| --- | --- | --- | --- |
| **Demographics** | | | |
| Age, years | 61.910 ± 10.068 | 61.290 ± 13.951 | 0.785 |
| Male, n (%) | 101 (79.5) | 49 (77.8) | 0.781 |
| BMI, kg/m^2^ | 24.390 [22.260-27.310] | 24.310 [21.630-26.290] | 0.109 |
| **Cardiovascular risk factors** | | | |
| Hypertension, n (%) | 82 (64.6) | 39 (61.9) | 0.719 |
| Diabetes, n (%) | 38 (29.9) | 23 (36.5) | 0.360 |
| Dyslipidemia, n (%) | 16 (12.6) | 12 (19.0) | 0.238 |
| Smoking history, n (%) | 63 (49.6) | 37 (58.7) | 0.236 |
| Alcohol consumption history, n (%) | 42 (33.1) | 19 (30.2) | 0.686 |
| **Clinical presentation** | | | |
| CCS, n (%) | 40 (31.5) | 21 (33.3) | 0.798 |
| ACS, n (%) | 87 (68.5) | 42 (66.6) | 0.798 |
| **Laboratory parameters** | | |  |
| TG, mmol/L | 1.410 [1.040-1.990] | 1.600 [1.090-2.450] | 0.329 |
| HDL-C, mmol/L | 0.970 [0.850-1.160] | 0.960 [0.830-1.130] | 0.676 |
| LDL-C, mmol/L | 2.420 [1.670-3.170] | 2.070 [1.280-3.060] | 0.031 |
| LVEF, % | 66.100 [59.800-70.300] | 63.300 [57.800-68.400] | 0.134 |
| HbA1c, % | 6.100 [5.700-6.900] | 6.100 [5.700-7.100] | 0.859 |
| Creatinine, μmol/L | 76.000 [66.000-88.000] | 74.000 [65.000-83.000] | 0.484 |

Continuous variables are presented as mean ± standard deviation or median [interquartile range], as appropriate, and categorical variables are presented as n (%). Included patients refer to the final study cohort. Excluded patients refer to those without follow-up coronary angiography. BMI, body mass index; CCS, chronic coronary syndrome; ACS, acute coronary syndrome; TG, triglyceride; HDL-C, high-density lipoprotein cholesterol; LDL-C, lowdensity lipoprotein cholesterol; LVEF, left ventricular ejection fraction; HbA1c, glycated hemoglobin.
